# Impact of Natural Heat Stress on Pregnant Rabbits: Behavioral, Physiological, and Reproductive Changes and the Ameliorative Role of Curcumin and Vitamin D_3_

**DOI:** 10.3390/vetsci13050412

**Published:** 2026-04-22

**Authors:** Mahmoud Roshdy, Hassan A. Khalil, Doaa E. Saad, Mahmoud Kamal, Mostafa A. Ayoub, Yasser Alrauji, Mohamed Shehab-El-Deen

**Affiliations:** 1 Animal Production Department, Faculty of Agriculture, Suez Canal University, Ismailia 41522, Egypt; mahmoud_roshdy@agr.suez.edu.eg (M.R.); hkhalil011971@agr.suez.edu.eg (H.A.K.);; 2 Animal Production Research Institute, Agricultural Research Center, Dokki, Giza 12618, Egypt; 3 Department of Animal and Poultry Production, College of Agriculture and Food, Qassim University, Buraydah 51452, Saudi Arabia

**Keywords:** rabbit does, pregnancy, thermotolerance, natural additives, antioxidants, physiology

## Abstract

This study evaluated the effects of heat stress on productive performance, physiology, reproduction, and oxidative status in pregnant New Zealand White (NZW) rabbit does, as well as the potential synergistic effects of curcumin and vitamin D3 (Cur + VD_3_) supplementation in alleviating these stress-induced impairments. Eighty multiparous does (12–18 months old) were assigned to a 2 × 2 factorial design involving two ambient temperatures (indoor vs. outdoor) and two supplementation levels (with or without Cur + VD_3_). Outdoor does experienced severe heat stress (THI = 33.22) compared to indoor thermal comfort conditions (THI = 25.13). The supplement (Cur + VD_3_) was administered orally at 1 mL/kg body weight. Heat stress significantly decreased body weight, milk yield, litter size, weight at weaning, and behavioral activity. Conversely, rectal temperature, respiration rate, and mortality increased. Supplementation with Cur + VD_3_ showed improved body weight, reproductive parameters, milk yield, and behavior, while reducing mortality (0% vs. 5%) compared to treatment without these additives. Physiologically, Cur + VD_3_ lowered rectal temperature and respiration rate. In conclusion, combined curcumin and vitamin D_3_ supplementation is an effective nutritional strategy to improve heat stress tolerance and maintain productivity in pregnant rabbits exposed to high ambient temperatures.

## Simple Summary

Heat stress in pregnant rabbits is a significant concern, as it can lead to adverse pregnancy outcomes such as increased mortality, reduced litter size, and lower birth weights. This study aims to examine the consequences of heat stress on productive efficiency, physiology, reproduction, and antioxidant levels in rabbits, as well as the potential synergistic benefits of curcumin and vitamin D_3_ in mitigating heat stress-induced damage. The results exhibited that heat stress reduced body weight, milk yield, litter size, and weaning weight, and altered behavior, while increasing rectal temperature, respiration rate, and mortality. The treatment with curcumin combined with vitamin D_3_ improved these parameters, decreased mortality, and enhanced heat tolerance and productivity. This approach offers a promising solution to the challenges associated with rabbit farming in hot climates.

## 1. Introduction

Climate change represents a significant global challenge, with rising ambient temperatures posing serious threats to farm animal productivity. Elevated temperatures induce heat stress, disrupting key metabolic and physiological processes, including thermoregulation, immune function, and reproductive and productive performance [1,2]. The hot climate in Egypt makes raising rabbits particularly difficult. For example, there are power outages during the day that can cause temperatures in rabbit housing to reach 32 °C or higher. This increases stress on rabbits, especially pregnant ones, leading to a high rate of abortion and early embryonic loss [3,4]. Prolonged exposure to high temperatures affects basal body temperature and heart rate and disrupts endocrine balance. Furthermore, heat stress stimulates the synthesis of heat shock proteins and activates inflammatory pathways, further exacerbating physiological strain and decreasing efficiency [4,5]. These changes induce cellular stress, resulting in the overproduction of reactive oxygen species (ROS). Heat stress-driven increased metabolism generates ROS that overwhelm the animal’s antioxidant defense mechanisms [6,7]. Oxidative stress damages lipids, proteins, and DNA, causing membrane instability, impaired enzyme function, mitochondrial dysfunction, ATP depletion, and apoptosis. Consequently, livestock exhibit reduced growth, compromised immunity, and organ dysfunction [8,9].

Rabbits are homeothermic animals. They typically regulate their body temperature through body positioning, increased breathing rate, and heat loss via vasodilation in the ears, which serve as major mechanisms for thermolysis [10]. The thermoneutral zone for rabbits ranges from 18 to 21 °C; however, in regions with elevated temperatures, typical conditions often exceed this range, resulting in significant heat stress [2]. This stress adversely affects productivity, metabolism, the physiological responses, reproductive functions, and overall homeostasis [5,8]. In female rabbits, exposure to heat stress leads to decreased conception rates, impaired embryonic development, reduced litter size and weight, and diminished milk production, while also increasing the age at puberty as well as pre- and post-weaning mortality [11]. Elevated temperatures disrupt hormonal balance and increase the concentration of ROS, which can cause oxidative damage to multiple organs [6,7]. Rabbits are particularly vulnerable due to their dense fur, limited behavioral thermoregulation, and prolonged exposure to high temperatures [12,13]. Improving environmental conditions is essential for rabbit welfare. Enhancing the quality of life for rabbits is critical, as it directly influences their welfare, health, and productivity [14]. Therefore, understanding the detrimental effects of heat stress on rabbits is vital; nutritional strategies should be considered to mitigate heat stress and improve their health [13].

Organic additives such as herbs and their derivatives help mitigate stress in animals, thereby promoting growth, enhancing immunity, and restoring oxidative balance [15,16]. Curcumin, a bioactive polyphenol extracted from turmeric, is recognized for its potent antioxidant, anti-inflammatory, and immunomodulatory properties [17,18]. In rabbits, dietary curcumin supplementation improves growth rates, feed conversion ratio (FCR), liver and kidney function, immune responses, and stress resistance, without negatively affecting feed intake (FI) or behavior [19,20,21]. Additionally, curcumin can enhance reproductive hormone levels and reduce oxidative stress by stimulating antioxidant enzyme activity [22,23].

In addition to phytochemicals, vitamins C, D_3_, and E are used to alleviate stress in livestock, promoting animal welfare by reducing physiological strain and environmental impact. Vitamin D3 (cholecalciferol) is essential for calcium and phosphorus metabolism, supporting bone development and nutrient absorption [24,25]. It enhances efficiency and growth in rabbits without significantly affecting body weight (BW), regulates mineral metabolism, and optimizes fertility [26,27]. Moreover, vitamin D3 provides cytoprotective and antioxidant benefits, helping to reduce oxidative stress and enhancing defense mechanisms [13,28].

Investigating the potential synergistic effects of plant components in combination with other therapeutic agents may open new avenues [29]. The interaction may occur through the combined use of both compounds, benefiting from their positive effects. Curcumin provides immediate ROS scavenging [30], while Vitamin D3 positively regulates antimicrobial activity and modulates inflammatory responses [31]. Therefore, it is hypothesized that combining these two compounds mitigates the negative effects of heat stress on pregnant rabbits. In this context, the present study aimed to evaluate the effects of heat stress on productive performance, physiology, reproduction, and oxidative status in rabbit does, as well as to evaluate the potential synergistic effects of curcumin and vitamin D3 supplementation in alleviating these stress-induced impairments.

## 2. Materials and Methods

### 2.1. Animal Diet and Management

The research was conducted at the Experimental Rabbitry Farm of the Faculty of Agriculture, Suez Canal University, Ismailia Governorate, Egypt, from July to September 2025 (summer). This experiment involved 80 New Zealand White (NZW) rabbit does that had previously given birth. The rabbits were between 12 and 18 months old, with an average weight of 2589 ± 45 g. At the start of the trial, all rabbits were healthy and free of external parasites or skin conditions. Each rabbit was housed individually in a galvanized wire cage measuring 50 × 50 × 40 cm. During the experiment, the animals had ad libitum access to food and water. They were maintained under identical environmental conditions, with a controlled photo period of 16 h of light and 8 h of darkness daily. The rabbits were fed a basal pelleted diet. The composition of the diet was formulated according to NRC [32] recommendations. The ingredients and proximate chemical analysis of the experimental diet are presented in Table 1.

### 2.2. Averages of Ambient Temperature (AT, °C), Relative Humidity (RH, %)

Ambient temperature and relative humidity were recorded daily in both indoor and outdoor rabbitries using a thermometer and a hygrometer. The temperature–humidity index (THI) was calculated according to Marai et al. [10] as an indicator of the thermal comfort level in the rabbit’s environment, based on ambient temperature and relative humidity.

It was calculated using the following equation: THI = db°C - [(0.31 -0.31 RH) × (db°C − 14.4)], where db°C is the dry bulb temperature in Celsius and Rh = RH%/100. The estimated THI values were classified as follows: less than 27.8 = absence of heat stress (thermal comfort zone), 27.8 to <28.9 = moderate heat stress, 28.9 to <30.0 = severe heat stress, and ≥30.0 = very severe heat stress.

### 2.3. Experimental Design

After a five-day acclimatization period, a 2 × 2 factorial experiment was conducted to evaluate the effects of ambient temperature conditions and oral supplementation with curcumin plus vitamin D_3_ (Cur + VD_3_) on the studied traits. Two factors were examined: temperature condition (indoor vs. outdoor) and oral Cur + VD_3_ supplementation (with or without). Natural mating was used to induce pregnancy, and conception was confirmed by abdominal palpation on day 10 post-mating.

A total of 80 multiparous pregnant NZW does were individually weighed and randomly allocated to four equal treatment groups (20 does per group): (1) indoor without commercial supplement (Cur + VD_3_), (2) indoor with Cur + VD_3_, (3) outdoor without Cur + VD_3_, and (4) outdoor with Cur + VD_3_. The supplement (Cur + VD_3_) was prepared as an aqueous suspension from a commercial dietary product (Biobnz Cur + VD_3_^®^, Biobnz Pharm, Cairo, Egypt) using glycerol and 70% sorbitol to enhance solubility. Each milliliter contained 3 mg of curcumin extract (equivalent to 0.18 mg curcuminoids) and 1.2 IU of vitamin D_3_. The supplement (Cur + VD_3_) was administered orally to the rabbits at a dosage of 1 mL/kg BW. Throughout the experimental period, all pregnant does were housed indoors under natural ambient conditions; however, does in groups 3 and 4 were temporarily moved outdoors and exposed to indirect solar radiation under shade for one hour daily (between 14:00 and 15:00) on days 15, 16, and 17 of pregnancy, respectively.

### 2.4. Live Body Weight, and Body Weight Changes

Each female rabbit was weighed individually to the nearest 1 g using a digital balance (MM-9003, Zhongshan, China) at mating, on day 15 post-mating (the onset of heat stress, which lasted for 3 days), three days after treatment (on day 18 post-mating, following heat stress), at three and six days post-recovery (days 21 and 24 post-mating), at parturition, and at weaning of the kits. Changes in female body weight (BW) from mating to parturition and from mating to kit weaning were subsequently calculated as follows:

BW change from mating to parturition = BW at parturition − BW at mating

BW change from mating to kit weaning = BW at kit weaning − BW at mating

### 2.5. Productive Performance

Litter size and litter weight at birth and at weaning were recorded for each doe in all experimental groups throughout the study. Milk yield was measured once per week for each doe in all experimental groups, between 08:00 and 09:00 during the first to the fourth week of lactation. This was performed by calculating the difference in kit weight before and after suckling. Before suckling, kits were separated from their does for 14 h during the first, second, third, and fourth weeks of lactation, following the method described by Khalil et al. [33].

### 2.6. Behavioral Activities, Traits, and Mortality Rate

Basic behaviors, including standing, walking, and sitting, were recorded for each doe in all experimental groups using a video recording system. Behavioral observations were conducted individually for each animal over one hour in the morning (between 09:00 and 11:00 a.m.) during the three days of treatment. Observations were made at 5 min intervals, and the number of animals exhibiting each behavior at each sampling point was recorded. The frequency of each behavior was expressed as the percentage of animals observed, following the method described by Khalil et al. [34]. Also, rabbits were observed daily to record mortality throughout the experimental period. The mortality rate was calculated as follows:

Mortality rate = (Number of dead rabbits/total number of rabbits at the beginning) × 100

### 2.7. Physiological Body Reactions

Thermo-respiratory responses, including rectal temperature (RT) and respiratory rate (RR), were recorded over six days for each doe in all experimental groups: three days during heat stress and three days during the subsequent recovery period. Respiratory rate was measured by counting thoracic movements (breaths per minute) for each animal, following the method described by Santangelo et al. [35]. Rectal temperature was measured using a clinical thermometer inserted approximately 2 cm into the rectum for one minute after lubrication with pure Vaseline, as outlined by Khalil et al. [33].

### 2.8. Statistical Analyses

Data were analyzed using the General Linear Model (GLM) procedure of SAS, 8.2 (2001) (SAS Institute Inc., Cary, NC, USA , 2001). Differences among means were determined using Duncan’s multiple range test [36], with the significance level set at *p* < 0.05. A two-way analysis of variance was conducted for all traits using the following model:

Y_ijk_ = μ + T_i_ + C_j_ + T_i_C_j_ + e_ijk_

where Y_ijk_ = the observation on the kth individual from the ith temperature conditions in jth curcumin + VD_3_; μ = the overall mean; T_i_ = the fixed effect of the ith temperature conditions (i = indoor and outdoor); C_j_ = the fixed effect of the jth curcumin + VD_3_ (j = with and without curcumin+ VD_3._); T_i_C_j_ = the interaction between ith temperature conditions and jth curcumin+ VD_3_; e_ijk_ = the random error associated with the Individual i_jk_.

## 3. Results

### 3.1. Temperature–Humidity Index (THI)

According to the equation of Marai et al. [11], the calculated THI values are presented in Table 2. The THI value under indoor rabbitry conditions was 25.13, indicating that the rabbits were maintained within the thermal comfort zone. In contrast, the THI value under outdoor conditions reached 33.22, reflecting that the rabbits were exposed to very severe heat stress.

### 3.2. Live Body Weight and Body Weight Change

The effects of temperature conditions (indoor vs. outdoor), oral administration of curcumin and vitamin D_3_ (Cur + VD_3_), and their interaction on body weight and body weight changes during different stages of the experiment are summarized in Table 3. The monitored time points included: at mating, at the start of treatment (15 days post-mating), 3 days post-treatment, 3 and 6 days post-recovery, parturition, at weaning, as well as the overall weight changes from mating to parturition and from mating to weaning.

Analysis of variance revealed significant differences (*p* < 0.05) in BW and body weight change across all evaluated stages, except at mating and at the beginning of treatment. The absence of statistical differences at these initial points confirms successful random allocation and baseline uniformity among treatment groups. Irrespective of Cur + VD_3_ supplementation, does housed under indoor conditions exhibited significantly greater body weight and body weight change at all evaluated stages compared to those kept outdoors. Similarly, Cur + VD_3_-supplemented animals consistently outperformed their non-supplemented counterparts in both parameters, regardless of temperature conditions.

The results showed a significant interaction effect between temperature and supplementation on body weight during various periods. As for interaction effects, three days after treatment, the highest body weight was observed in does kept under outdoor conditions and receiving Cur + VD_3_ supplementation, whereas the lowest body weight was recorded in outdoor-housed animals without Cur + VD_3_ supplementation (*p* = 0.042). This trend persisted throughout the experimental period, with outdoor-housed, non-supplemented does showing significantly lower body weight and body weight changes compared to all other treatment groups.

### 3.3. Reproductive Traits of Rabbit Does

During the study, all does successfully kindled, and no pregnancy losses occurred before term. The effects of temperature conditions (indoor vs. outdoor), oral administration of curcumin and vitamin D3 (Cur + VD_3_), and their interaction on the reproductive performance of rabbit does—including litter size and weight at birth and weaning, livability rates, and average kit weight—are presented in Table 4. Analysis of variance revealed significant differences (*p* < 0.05) in all measured reproductive traits due to the main effects and their interaction, except for litter size at birth and average kit weight at weaning between temperature treatments, regardless of Cur + VD_3_ supplementation. Does housed under indoor conditions showed significantly (*p* < 0.05) enhanced reproductive outcomes in all parameters except litter size at birth and average kit weight at weaning, irrespective of Cur + VD_3_ administration. Similarly, Cur + VD_3_-treated females exhibited significant improvements in all traits, excluding litter size at birth, independent of housing temperature.

Regarding the interaction effect, does kept under outdoor conditions without Cur + VD_3_ supplementation demonstrated significant reductions in all reproductive traits compared to the other groups, except for litter size at birth, which remained unaffected.

### 3.4. Milk Yield of Rabbit Does

The average weekly milk yield, as affected by the main factors and their interaction, is presented in Table 5. Analysis of variance revealed a significant effect of temperature treatment only during the fourth week of the lactation period. In contrast, the Cur + VD_3_ treatment and its interaction with temperature showed significant effects throughout all studied weeks and the overall lactation period. During the fourth week of lactation, indoor-housed animals exhibited significantly higher milk yield compared with outdoor-housed animals, irrespective of Cur + VD_3_ administration.

In contrast, animals supplemented with Cur + VD_3_ exhibited higher (*p* < 0.05) milk yield throughout all studied periods of lactation, irrespective of temperature treatment. Animals housed indoors produced significantly more milk (*p* < 0.05) than those kept under outdoors during the fourth week of lactation, irrespective of Cur + VD_3_ supplementation. Furthermore, animals receiving Cur + VD3 supplementation showed significantly higher milk yield across all studied weeks of the lactation period, regardless of temperature treatment. Regarding interaction effects, animals supplemented with Cur + VD_3_ and housed under both indoor and outdoor conditions exhibited significantly higher milk yield compared to those housed under the same conditions without Cur + VD_3_ supplementation during the first, second, and third weeks, as well as over the entire lactation period. Additionally, does kept outdoors without Cur + VD_3_ supplementation had significantly lower milk yield than all other treatment groups during the fourth week of lactation.

### 3.5. Physiological Body Reactions of Rabbit Dose

The effects of different treatments and their interactions on physiological responses—specifically rectal temperature (°C) and respiration rate (breaths per minute)—during the treatment and recovery periods are presented in Table 6 and Table 7. ANOVA revealed significant differences among the main effects and their interactions across all measured physiological parameters throughout both phases. However, no significant differences were observed on the first day of treatment among animals administered Cur + VD_3_, regardless of ambient temperature conditions.

Animals exposed to outdoor conditions exhibited significantly higher (*p* < 0.05) rectal temperatures and respiration rates compared to those housed in indoor conditions during all three treatment days and throughout the recovery period, except on the third day of recovery, regardless of Cur + VD_3_ supplementation. Furthermore, rabbits administered Cur + VD_3_ exhibited significantly reduced (*p* < 0.05) rectal temperatures and respiration rates compared to untreated animals during both the treatment and recovery phases, except on the first treatment day.

Regarding interaction effects, animals housed outdoors exhibited significantly higher rectal temperatures on the first day post-treatment compared to those kept indoors, regardless of Cur + VD_3_ treatment. Moreover, animals exposed to outdoor temperatures without Cur + VD_3_ supplementation showed significantly higher rectal temperatures than all other experimental groups during the second and third days of treatment, as well as throughout the entire recovery period. Similarly, animals maintained outdoors without Cur + VD_3_ supplementation exhibited significantly elevated respiration rates throughout the treatment period compared to all other groups. Conversely, during the recovery period, the lowest respiration rates were consistently observed in animals housed indoors and supplemented with curcumin and vitamin D3.

### 3.6. Basic Behavioral Activity Traits and Mortality Rate of Rabbit Does

The averages of basic behavioral activity traits, as influenced by the treatments and their interactions, are presented in Table 8. Analysis of variance revealed significant differences among the main factors as well as their interactions for all basic behavioral traits. Animals housed under indoor conditions exhibited significantly longer standing and walking times and significantly shorter sitting times compared to those kept under outdoor conditions, regardless of Cur + VD_3_ supplementation. Additionally, animals receiving Cur + VD_3_ supplementation showed significantly (*p* < 0.05) increased standing and walking durations, along with reduced sitting time, compared to non-supplemented animals, irrespective of temperature conditions.

The interaction between temperature and Cur + VD_3_ treatments revealed that animals housed indoors and supplemented with Cur + VD_3_ exhibited the highest percentages of standing and walking behaviors, along with the lowest percentage of sitting. In contrast, animals kept outdoors without Cur + VD_3_ supplementation showed the lowest time spent standing and walking, and the highest time spent sitting. Additionally, the mortality rate was also influenced by the treatments and their interactions. The indoor group had no mortality (0.00%), while the outdoor group had a mortality rate of 5.00% (*p* = 0.042). Among rabbits not receiving Cur + VD3, 5.00% died, whereas no deaths occurred in the supplemented groups (0.00%) (*p* = 0.042). Regarding the interaction, the highest mortality rate (10.00%) was observed in the outdoor (stressed) group without supplementation (*p* = 0.042).

## 4. Discussion

Heat stress adversely affected the physiological performance and productivity of rabbits. However, supplementation with curcumin and vitamin D_3_ mitigated these effects, enhancing heat tolerance and overall production outcomes. Heat stress causes animals to eat less, weakens their immune systems, and increases mortality rates, all of which result in significant financial losses [5]. In rabbits, heat stress is linked to oxidative imbalance, immune dysfunction, and endocrine disturbances, reduced productivity, and impaired reproduction [13]. Elevated temperatures lead to increased oxidative stress, producing excessive ROS and reactive nitrogen species. This weakens the body’s defenses against free radicals and disrupts normal cellular functions [6,8]. Rabbits are particularly vulnerable because they retain heat very effectively. In the current study, rabbits housed outdoors exhibited significant HS, with THI readings of 33.22—exceeding the critical threshold of 28.9—and nearly fatal rectal temperatures. As an indication group, the non-supplemented group displayed average values for each attribute. In contrast, rabbits supplemented with curcumin and vitamin D3 demonstrated significantly higher efficiency in physiological, reproductive, and productive parameters. The observed improvements in rabbits supplemented with curcumin and vitamin D_3_ demonstrate that these interventions effectively alleviate heat stress. This supports the study’s objective and confirms the hypothesis that dietary supplementation can enhance heat tolerance and productivity under high-temperature conditions.

This group’s exceptional FI and greatest BW changes indicate a state of maximum growth stimulation and optimal health. These results are aligned with those of Eissa et al. [37], who found that rabbits receiving combined curcumin and vitamin D3 treatment exhibited the highest cumulative growth rate. The synergistic interaction between curcumin and vitamin D3 accounts for the significant improvement in growth efficiency. Vitamin D3 promotes calcium absorption and cell membrane–associated enzyme activity, all of which contribute to effective cellular metabolism and tissue growth. Conversely, curcumin enhances enzyme production in the intestines and hepatopancreas, thereby improving nutrient breakdown, absorption, and utilization [37]. Furthermore, the potent antioxidant and anti-inflammatory properties of Cur decrease hepatic tissue damage, further improving metabolic efficiency and nutrient utilization [17,19]. It has been demonstrated that vitamin D3 primarily promotes growth by enhancing nutrient utilization rather than directly increasing growth rate. Although it has little effect on final body weight, earlier studies reported improvements in FCR, digestibility, plasma phosphorus levels, mortality rate, and skeletal development [25,27]. VD_3_ is essential for protein metabolism, muscle growth, and maintaining calcium–phosphorus balance; however, excessive intake can lead to hypercalcemia and metabolic toxicity, underscoring the importance of carefully monitored supplementation [25,38].

As a result of a forceful metabolic adaptation aimed at reducing endogenous heat production, pregnant rabbit does in the non-supplemented heat-stressed group (OD × −CV) in the current study showed a decrease in body weight. These findings are consistent with those of [12,39] and Abdelnour et al. [40], who showed that heat stress significantly impairs rabbit growth capacity and feed efficiency, mainly due to disruptions in metabolism, hormonal balance, and digestion. It has been demonstrated that exposure to temperatures above the thermoneutral zone (15–25 °C), particularly between 31 and 36 °C, substantially reduces BW, average daily gain, and FCR. Additionally, rabbits are especially vulnerable to these effects due to their dense fur, limited thermoregulatory ability, and lack of sweat glands. In warmer climates, heat stress decreases meat production and profitability by disrupting physiological homeostasis, increasing oxidative stress, and exacerbating growth depression [41,42]. A significant decrease in FI is one of the main mechanisms underlying growth restriction. When ambient temperatures exceed 26–28 °C, heat stress activates hypothalamic satiety centers, which lowers the heat increment of feeding (HIF)—the internal heat generated through digestion—and causes voluntary reductions in FI up to 50–65% [13] . Additionally, seasonal investigations show that FI decreases by 3% to 38% at 21 °C, directly limiting nutrient availability for changes in BW [11,43].

However, by regulating metabolism, vitamin D3 and curcumin supplements can reduce the growth inhibition caused by HS in rabbits. Particularly during periods of stress or illness, curcumin preserves intestinal structure, reduces oxidative damage, enhances liver function, and improves nutrient absorption and feed efficiency [19,37]. According to Gürer et al. [24], VD_3_ improves feed efficiency, and fertility while lowering lipid peroxidation and apoptosis. Additionally, it regulates calcium–phosphorus metabolism, skeletal growth, conception, and antioxidant defense.

Furthermore, heat stress adversely affects rabbits’ ability to reproduce and the survival of their progeny. Litter size, birth and weaning weights, survival rates, and milk production all significantly decreased in the non-supplemented outdoor group (OD × −CV). The negative impacts of heat stress on lactation were further confirmed by outbred rabbits, which exhibited a marked reduction in milk yield [44]. Pregnancy rates, embryo viability, litter size, and overall reproductive efficiency are all negatively impacted by HS. Under severe heat conditions, conception rates can decline from 100% to between 33% and 66% [13,45]. High ambient temperatures (25–36 °C) also decrease litter weights at birth to 41.5–56.6 g, compared to 52.7–61.4 g under thermoneutral conditions, while weaning weights decline from 630.5 g to 503.0 g [12,46]. These effects may be compounded by hormonal imbalances, including suppressed estrogen and progesterone levels, disrupted estrus cycles, and abnormal ova morphology, alongside reduced uterine and placental blood flow, all of which may exacerbate reproductive failure [13].

In the current study, rabbits supplemented with Cur + VD3 had higher milk yield at all studied periods of lactation. The previous studies showed that dietary supplementation with curcumin or turmeric extracts can enhance milk yield by improving mammary gland function, milk composition, and reducing oxidative stress during lactation. These effects are partly attributed to their antioxidant, anti-inflammatory, and metabolic properties, including increased prolactin levels [37,47]. The reproductive effects of curcumin are dose-dependent: at high concentrations, it may modulate ovarian function adversely, harm blastocysts, or increase the risk of spontaneous abortion, whereas protective effects are observed in pathological pregnancies through reduced placental inflammation and oxidative stress [48,49]. Vitamin D3 is also essential for female reproduction, while deficiencies reduce fertility and the viability of offspring.

Rabbit welfare under environmental stress is influenced by multiple factors. Housing and environmental conditions significantly affect rabbits’ stress responses, abnormal behaviors, and productive outcomes [14]. Likewise, rabbits exposed to heat stress exhibit notable physiological and behavioral changes. For example, the OD-CV group showed a substantial increase in heart rate (breaths per minute) and rectal temperature compared to other groups. Due to their thick fur and lack of sweat glands, rabbits often experience core and rectal body temperatures rising above 41 °C, and in severe outdoor heat stress cases, temperatures can exceed 43.5 °C, indicating impaired thermoregulation [39,42]. Rectal temperature is a sensitive indicator of heat stress, as even slight increases (<1 °C) can negatively impact welfare and productivity [12,50]. Curcumin intake reduces inflammation and has an antipyretic effect, stabilizing rectal temperature [51]. Vitamin D3 promotes thermoregulation, possibly by enhancing nutrient utilization, improving metabolic efficiency, and modulating inflammatory responses, thereby increasing resistance to heat stress [5,31].

Due to their heavy reliance on respiratory evaporative cooling, rabbits’ respiration rates increase significantly in hotter environments, rising from 69 to 190 breaths per minute. Outdoor rabbits can reach approximately 184.88 breaths per minute [11]. Although this energy-intensive process is initially effective, it can generate additional metabolic heat and lead to unbalanced respiration and respiratory alkalosis [52]. Vitamin D3 may enhance cell viability and reduce oxidative stress [53], thereby improving respiratory function under heat stress. Joshi et al. [22] showed that curcumin (80 mg/kg diet) significantly reduced elevated rectal temperature in rabbits for several hours after treatment, indicating that curcumin has antipyretic activity comparable to that of paracetamol. Furthermore, curcumin has been demonstrated to improve markers of oxidative stress, oxygenation parameters, and lung tissue preservation in rabbit models of pulmonary injury, despite the lack of direct data on its impact on respiration rate [54]. Also, Ramirez-Tortosa et al. [55] revealed that curcumin administration led to increased mitochondrial antioxidant activity, decreased mitochondrial reactive oxygen species, and improved mitochondrial function in rabbits. Based on these findings, curcumin may positively influence the improvement of respiration rates in rabbits.

In terms of behavior, the current study found that rabbits exposed to heat stress spent more time sitting and less time standing or walking compared to those in other groups. Additionally, prone or stretched lying and ear spreading are thermoregulatory postures induced by HS that improve air circulation and simultaneously decrease activity levels to conserve energy [13,42]. Under extreme heat conditions, when homeostatic processes fail, rabbits may exhibit a “stretched sitting and energy-saving adaptation [39]. In the current study, curcumin administration did not significantly change overall behavior, indicating that it is safe and compatible with the body’s natural thermoregulatory mechanisms. Conversely, vitamin D3 indirectly supports behavioral adaptation by enhancing antioxidant and metabolic capacity, helping rabbits maintain activity levels during heat stress [37,56].

In the current study, treatments significantly affected survival and mortality rates; the 10% mortality rate in the OD X-CV group compared to 0% in the +CV group underscores the lethality of excessive heat for rabbits. While specific data on the effects of curcumin combined with vitamin D3 under heat stress on mortality are limited, both compounds improve overall vitality and reduce damage associated with oxidative stress. This supports that curcumin is a viable natural method for enhancing stress tolerance in rabbit husbandry.

Every study has its limitations, which are essential for future research. Future studies can improve upon the findings of this study by using longer exposure periods, larger sample sizes, more comprehensive assessments of physiological and oxidative stress markers, and a wider range of behavioral measurements, which will help provide a clearer picture of the observed effects. These efforts will contribute to a more thorough understanding of the study subject.

## 5. Conclusions

The results of this study show that the combined supplementation with curcumin and vitamin D3 may mitigate some adverse effects associated with heat stress, such as weight loss, reproductive problems, and oxidative damage. The combined supplements work synergistically to boost the body’s natural responses and improve metabolic function. Additionally, curcumin and vitamin D_3_ help the body return to normal function under heat stress, making supplemented rabbits more resilient than those without supplementation. Further studies are needed to confirm the efficacy, determine the optimal dosage, and elucidate the mechanism of action under more robust heat stress models.

## Figures and Tables

**Table 1:** Ingredients and calculated chemical composition of the basal diet.

Ingredients	(%)
Alfalfa hay	26.5
Barley	17.0
Yellow corn	15.0
Soybean meal (44%)	16.0
Wheat bran	20.0
Alfalfa straw	3.0
Limestone	1.65
Vitamins and minerals premix *	0.30
NaCl	0.30
dl- methionine	0.1
Anti-coccidia	0.05
Anti-toxin	0.1
Total	100.0
Chemical composition (as DM basics).	
Crude protein %	17.5
Digestible energy (kcal/kg)	2500
Crude fiber %	12.6
Calcium %	0.93
Total phosphorus %	0.62
Lysine %	0.84
Methionine %	0.65
Methionine + Cysteine %	0.63

* Supplied per 1 kg diet: 6000 IU vit. A; 900 IU vit. D3; 40 mg vit. E; 2.0 mg vit. K3; 2.0 mg vit. B1; 4.0 mg vit. B2; 2.0 mg vit. B6; 0.010 mg vit. B12; 5.0 mg vit. PP; 10.0 mg vit. B5; 0.05 mg B8; 3.0 mg B9; 250 mg choline; 50.0 mg Fe; 50.0 mg Zn; 8.5 mg Mn; 5.0 mg Cu; 0.20 mg I, and 0.15 mg Se.

**Table 2:** Averages of ambient temperature (AT, °C), relative humidity (RH, %), and temperature-humidity index (THI) indoor and outdoor in the rabbitry during the experimental periods.

Housing	AT (°C)			RT (%)	THI
	Max	Min	Mean		
Indoor conditions	31.8 ± 0.5	22.3 ± 0.9	27.05 ± 0.8	54.2 ± 5.7	25.13
Outdoor conditions	39.7 ± 1.5	37.8 ± 0.8	38.75 ± 0.7	27.4 ± 3.7	33.22

**Table 3:** Average live body weight (g) and body weight changes at different measurement stages as affected by treatments (Mean ± SE).

Main Effects	At Mating	15 Days Post-Mating (Starting Experiment)	3 Days Post Treatment	3 Days Post Recovery	6 Days Post Recovery	At Parturition	At Weaning	Changes from Mating to Parturition	Changes from Mating to Weaning
Temperature (ID, OD)									
ID	2617.50 ± 39.40	3070.00 ± 61.17	3140.50 ^a^ ± 59.68	3291.50 ^a^ ± 56.08	3525.00 ^a^ ± 58.45	3153.00 ^a^ ± 53.35	3324.00 ^a^ ± 49.07	535.50 ^a^ ± 30.79	706.50 ^a^ ± 33.52
OD	2557.89 ± 50.95	3115.78 ± 70.61	3056.31 ^b^ ± 79.27	3097.36 ^b^ ± 92.83	3282.63 ^b^ ± 99.10	2926.31 ^b^ ± 90.03	3115.78 ^b^ ± 92.04	368.42 ^b^ ± 63.33	557.89 ^b^ ± 68.80
*p *value	0.358	0.626	0.047	0.048	0.040	0.035	0.050	0.021	0.050
S upplementation** (-CV, +CV)**									
-CV	2615.26 ± 43.05	3063.15 ± 64.07	3013.15 ^b^ ± 68.48	3048.94 ^b^ ± 81.7280	3215.78 ^b^ ± 84.56	2896.31 ^b^ ± 80.94	3045.78 ^b^ ± 75.34	281.05 ^b^ ± 45.91	430.52 ^b^ ± 42.02
+CV	2563.00 ± 47.35	3120.00 ± 67.12	3181.500 ^a^ ± 66.7173	3337.500 ^a^ ± 61.0085	3588.50 ^a^ ± 61.35	3181.50 ^a^ ± 58.97	3390.50 ^a^ ± 54.67	618.50 ^a^ ± 21.37	827.50 ^a^ ± 17.90
*p *value	0.421	0.545	0.049	0.007	0.001	0.007	0.001	0.000	0.000
Interactions									
ID × -CV	2670.00 ± 56.45	3070.00 ± 93.15	3120.00 ^ab^ ± 93.74	3253.00 ^a^ ± 87.54	3440.00 ^a^ ± 86.90	3106.00 ^a^ ± 83.57	3249.00 ^a^ ± 74.27	436.00 ^b^ ± 32.46	579.00 ^b^ ± 25.44
ID × +CV	2565.00 ± 52.51	3070.00 ± 84.39	3161.00 ^ab^ ± 78.47	3330.00 ^a^ ± 72.72	3610.00 ^a^ ± 72.57	3200.00 ^a^ ± 67.39	3399.00 ^a^ ± 58.33	635.00 ^a^ ± 27.37	834.00 ^a^ ± 22.02
OD × -CV	2554.44 ± 62.78	3055.55 ± 92.96	2894.44 ^b^ ± 89.14	2822.22 ^b^ ± 99.69	2966.66 ^b^ ± 98.93	2663.33 ^b^ ± 97.45	2820.00 ^b^ ± 89.20	108.88 ^c^ ± 40.70	265.55 ^c^ ± 33.42
OD × +CV	2561.00 ± 81.91	3170.00 ± 106.51	3202.00 ^a^ ± 111.99	3345.00 ^a^ ± 102.04	3567.00 ^a^ ± 102.60	3163.00 ^a^ ± 100.33	3382.00 ^a^ ± 95.93	602.00 ^a^ ± 33.45	821.00 ^a^ ± 29.30
*p *value	0.537	0.815	0.042	0.001	0.000	0.000	0.000	0.000	0.000

^a,b,c^ Means within the same column not sharing a common superscript differed significantly; ID: Indoor; OD: Outdoor; -CV: Without Curcumin + Vit D3; +CV: With Curcumin + Vit D3 supplementation.

**Table 4:** Reproductive performance of rabbit does as affected by treatments (Mean ± SE).

Main Effects	Litter Size at Birth	No of Live Kits at Birth	Livability at Birth	Litter Weight at Birth	Average Kit Weight at Birth	Litter Size at Weaning	Livability at Weaning	Litter Weight at Weaning	Average Kit Weight at Weaning
Temperature (ID, OD)									
ID	8.95 ± 0.33	8.20 ^a^ ± 0.27	92.44 ^a^ ± 2.33	387.25 ^a^ ± 7.48	44.34 ^a^ ± 1.74	7.20 ^a^ ± 0.31	88.52 ^a^ ± 3.32	1807.10 ^a^ ± 72.51	250.12 ± 8.56
OD	9.10 ± 0.26	7.21 ^b^ ± 0.46	79.43 ^b^ ± 4.69	352.36 ^b^ ± 11.93	39.11 ^b^ ± 1.53	4.52 ^b^ ± 0.64	59.68 ^b^ ± 8.08	1353.62 ^b^ ± 172.32	244.61 ± 8.67
*p *value	0.720	0.048	0.016	0.017	0.031	0.010	0.002	0.005	0.451
S upplementation (-CV, +CV)									
-CV	9.05 ± 0.32	6.80 ^b^ ± 0.40	76.29 ^b^ ± 4.30	335.52 ^b^ ± 8.45	38.03 ^b^ ± 1.89	4.15 ^b^ ± 0.55	59.01 ^b^ ± 8.27	1158.18 ^b^ ± 135.01	232.13 ^b^ ± 8.81
+CV	9.00 ± 0.28	8.50 ^a^ ± 0.28	94.79 ^a^ ± 2.18	403.25 ^a^ ± 5.78	45.38 ^a^ ± 1.11	7.55 ^a^ ± 0.29	89.15 ^a^ ± 2.52	1963.45 ^a^ ± 46.27	264.09 ^a^ ± 3.41
*p *value	0.90	0.002	0.001	0.000	0.001	0.000	0.051	0.000	0.002
Interactions									
ID × -CV	9.00 ± 0.53	7.90 ^a^ ± 0.37	89.05 ^a^ ± 3.50	364.50 ^b^ ± 5.89	42.02 ^a^ ± 3.00	6.40 ^b^ ± 0.33	82.54 ^a^ ± 5.44	1538.20 ^c^ ± 57.04	240.34 ^a^ ± 12.46
ID × +CV	8.90 ± 0.43	8.50 ^a^ ± 0.40	95.83 ^a^ ± 2.84	410.00 ^a^ ± 9.30	46.67 ^a^ ± 1.62	8.00 ^a^ ± 0.39	94.50 ^a^ ± 3.02	2076.00 ^a^ ± 53.64	259.50 ^a^ ± 11.59
OD × -CV	9.11 ± 0.38	5.71 ^b^ ± 0.54	63.53 ^b^ ± 5.47	303.33 ^c^ ± 7.07	33.59 ^b^ ± 1.02	1.66 ^c^ ± 0.42	32.87 ^b^ ± 11.20	524.83 ^d^ ± 87.77	209.93 ^b^ ± 3.19
OD × +CV	9.100 ± 0.37	8.50 ^a^ ± 0.42	93.75 ^a^ ± 3.44	396.50 ^a^ ± 6.68	44.08 ^a^ ± 1.50	7.10 ^ab^ ± 0.40	83.81 ^a^ ± 3.38	1850.90 ^b^ ± 57.86	260.69 ^a^ ± 8.77
*p *value	0.985	0.000	0.000	0.000	0.000	0.000	0.000	0.000	0.004

^a,b,c,d^ Means within the same column not sharing a common superscript differed significantly; ID: Indoor; OD: Outdoor; -CV: Without Curcumin + Vit D3; +CV: With Curcumin + Vit D3 supplementation.

**Table 5:** Average weekly milk yield (g/day/doe) as affected by treatments (Mean ± SE).

Main Effects	1st Week	2nd Week	3rd Week	4th Week	Overall
Temperature (ID, OD)					
ID	112.15 ± 2.40	101.75 ± 3.75	106.35 ± 4.74	113.65 ^a^ ± 3.98	108.47 ± 2.74
OD	116.05 ± 7.79	94.78 ± 4.16	105.87 ± 5.74	101.37 ^b^ ± 3.29	103.57 ± 3.72
*p *value	0.628	0.221	0.949	0.028	0.293
S upplementation (-CV, +CV)					
-CV	102.94 ^b^ ± 1.83	86.89 ^b^ ± 3.34	89.31 ^b^ ± 4.28	101.68 ^b^ ± 3.65	94.90 ^b^ ± 1.99
+CV	124.60 ^a^ ± 6.76	109.25 ^a^ ± 2.83	119.60 ^a^ ± 3.21	113.40 ^a^ ± 3.82	116.71 ^a^ ± 2.19
*p *value	0.005	0.000	0.000	0.032	0.000
Interactions					
ID × -CV	104.10 ^b^ ± 2.16	90.70 ^b^ ± 4.86	91.00 ^b^ ± 4.87	107.50 ^a^ ± 3.59	98.32 ^b^ ± 1.67
ID × +CV	120.20 ^a^ ± 2.31	112.80 ^a^ ± 2.92	121.70 ^a^ ± 4.36	119.80 ^a^ ± 6.67	118.62 ^a^ ± 2.46
OD × -CV	101.66 ^b^ ± 3.11	82.66 ^b^ ± 4.38	86.50 ^b^ ± 8.54	92.00 ^b^ ± 6.15	91.11 ^b^ ± 3.46
OD × +CV	129.00 ^a^ ± 3.21	105.70 ^a^ ± 4.54	117.50 ^a^ ± 4.85	107.00 ^a^ ± 2.63	114.80 ^a^ ± 3.67
*p *value	0.035	0.000	0.000	0.010	0.000

^a,b,^ Means within the same column not sharing a common superscript differed significantly; ID: Indoor; OD: Outdoor; -CV: Without Curcumin + Vit D3; +CV: With Curcumin + Vit D3 supplementation.

**Table 6:** Average rectal temperature (°C) as affected by treatments (Mean ± SE).

Main Effects	Duration of Treatment (days)			Duration of Recovery (days)		
	1st Day	2nd Day	3rd Day	1st Day	2nd Day	3rd Day
Temperature (ID, OD**)**						
ID	39.26 ^b^ ± 0.06	38.96 ^b^ ± 0.08	38.84 ^b^ ± 0.10	38.95 ^b^ ± 0.09	38.95 ^b^ ± 0.11	38.67 ^b^ ± 0.08
OD	41.44 ^a^ ± 0.17	41.76 ^a^ ± 0.27	41.81 ^a^ ± 0.27	39.32 ^a^ ± 0.09	39.30 ^a^ ± 0.09	39.13 ^a^ ± 0.09
*p *value	0.000	0.000	0.000	0.048	0.041	0.003
S upplementation (-CV, +CV)						
−CV	40.39 ± 0.20	41.15 ^a^ ± 0.36	41.14 ^a^ ± 0.35	39.56 ^a^ ± 0.06	39.47 ^a^ ± 0.05	39.28 ^a^ ± 0.08
+CV	40.30 ± 0.18	39.58 ^b^ ± 0.13	39.40 ^b^ ± 0.15	38.70 ^b^ ± 0.04	38.58 ^b^ ± 0.05	38.53 ^b^ ± 0.05
*p *value	1.000	0.003	0.000	0.000	0.000	0.000
Interactions						
ID × -CV	39.25 ^b^ ± 0.05	39.10 ^c^ ± 0.12	39.17 ^c^ ± 0.09	39.29 ^b^ ± 0.08	39.22 ^b^ ± 0.06	38.98 ^c^ ± 0.04
ID × +CV	39.27 ^b^ ± 0.11	38.83 ^c^ ± 0.11	38.51 ^d^ ± 0.11	38.62 ^c^ ± 0.09	38.58 ^c^ ± 0.15	38.37 ^d^ ± 0.08
OD × -CV	41.54 ^a^ ± 0.22	43.20 ^a^ ± 0.29	43.33 ^a^ ± 0.16	39.87 ^a^ ± 0.03	39.72 ^a^ ± 0.04	39.58 ^a^ ± 0.13
OD × +CV	41.34 ^a^ ± 0.15	40.33 ^b^ ± 0.12	40.29 ^b^ ± 0.11	38.78 ^c^ ± 0.06	38.59 ^c^ ± 0.05	38.69 ^b^ ± 0.09
*p *value	0.000	0.000	0.000	0.000	0.000	0.000

^a,b,c,d^ Means within the same column not sharing a common superscript differed significantly; ID: Indoor; OD: Outdoor; -CV: Without Curcumin + Vit D3; +CV: With Curcumin + Vit D3 supplementation.

**Table 7:** Average respiration rate (breaths/min) as affected by treatments (Mean ± SE).

Main Effects	Duration of Treatment (days)			Duration of Recovery (days)		
	1st Day	2nd Day	3rd Day	1st Day	2nd Day	3rd Day
Temperature (ID, OD)						
ID	150.00 ^b^ ± 0.68	149.80 ^b^ ± 0.84	145.20 ^b^ ± 1.64	144.00 ^b^ ± 1.98	144.40 ^b^ ± 1.98	139.80 ± 2.90
OD	169.20 ^a^ ± 1.92	172.80 ^a^ ± 2.01	174.84 ^a^ ± 2.09	152.71 ^a^ ± 0.48	151.46 ^a^ ± 0.58	143.68 ± 1.31
*p *value	0.002	0.000	0.000	0.000	0.000	0.204
S upplementation (-CV, +CV)						
-CV	163.60 ^a^ ± 2.45	167.20 ^a^ ± 2.59	167.15 ^a^ ± 2.89	153.05 ^a^ ± 0.51	152.84 ^a^ ± 0.44	150.94 ^a^ ± 0.46
+CV	155.60 ^b^ ± 1.23	155.40 ^b^ ± 1.43	152.00 ^b^ ± 2.03	143.60 ^b^ ± 1.45	143.00 ^b^ ± 1.32	132.60 ^b^ ± 1.44
*p *value	0.041	0.003	0.001	0.002	0.000	0.000
Interactions						
ID × -CV	150.40 ^c^ ± 0.88	152.40 ^c^ ± 0.93	151.20 ^c^ ± 0.99	152.00 ^a^ ± 1.03	152.40 ^ab^ ± 0.93	152.00 ^a^ ± 0.84
ID × +CV	149.60 ^c^ ± 1.06	147.20 ^d^ ± 0.80	139.20 ^d^ ± 1.55	136.00 ^b^ ± 1.19	136.40 ^c^ ± 1.25	127.60 ^c^ ± 1.39
OD × -CV	176.80 ^a^ ± 1.06	182.00 ^a^ ± 0.85	184.88 ^a^ ± 0.70	154.22 ^a^ ± 0.70	153.33 ^a^ ± 0.66	149.77 ^a^ ± 0.70
OD × +CV	161.60 ^b^ ± 0.88	163.60 ^b^ ± 1.39	164.80 ^b^ ± 0.80	151.20 ^a^ ± 0.99	149.60 ^b^ ± 1.22	137.60 ^b^ ± 2.32
*p *value	0.000	0.000	0.000	0.000	0.000	0.000

^a,b,c,d^ Means within the same column not sharing a common superscript differed significantly; ID: Indoor; OD: Outdoor; -CV: Without Curcumin + Vit D3; +CV: With Curcumin + Vit D3 supplementation.

**Table 8:** Basic behavioral activity % as affected by treatments (Mean ± SE).

Main Effects	Standing	Walking	Sitting	Mortality Rate (%)
Temperature (ID, OD)				
ID	30.50 ^a^ ± 4.16	32.50 ^a^ ± 3.13	37.00 ^b^ ± 4.63	0.00 ^b^ ± 0.00
OD	20.50 ^b^ ± 3.49	18.00 ^b^ ± 3.35	61.50 ^a^ ± 6.02	5.00 ^a^ ± 2.20
*p *value	0.003	0.003	0.048	0.000
S upplementation (-CV, +CV)				
-CV	16.00 ^b^ ± 3.06	19.50 ^b^ ± 3.60	64.50 ^a^ ± 5.68	5.00 ^a^ ± 2.20
+CV	35.00 ^a^ ± 3.64	31.00 ^a^ ± 3.17	34.00 ^b^ ± 4.09	0.00 ^b^ ± 0.00
*p *value	0.000	0.022	0.000	0.000
Interactions				
ID × -CV	23.50 ^b^ ± 4.41	28.50 ^b^ ± 4.34	48.00 ^b^ ± 6.28	0.00 ^b^ ± 0.00
ID × +CV	37.50 ^a^ ± 6.55	36.50 ^a^ ± 4.34	26.00 ^c^ ± 4.93	0.00 ^b^ ± 0.00
OD × -CV	8.50 ^c^ ± 2.79	10.50 ^c^ ± 4.24	81.00 ^a^ ± 6.04	10.00 ^a^ ± 6.11
OD × +CV	32.50 ^b^ ± 3.43	25.50 ^b^ ± 4.11	42.00 ^b^ ± 5.68	0.00 ^b^ ± 0.00
*p *value	0.000	0.001	0.000	0.042

^a,b,c^ Means within the same column not sharing a common superscript differed significantly; ID: Indoor; OD: Outdoor; -CV: Without Curcumin + Vit D3; +CV: With Curcumin + Vit D3 supplementation.

## Data Availability

The original contributions presented in this study are included in the article. Further inquiries can be directed to the corresponding author(s).

## Abbreviations

The following abbreviations are used in this manuscript:

NZW	New Zealand White
Cur	curcumin
VD_3_	vitamin D_3_
THI	temperature–humidity index
RH	relative humidity
AT	ambient temperature
dbt	dry bulb temperature
RT	rectal temperature
